# Verbal Communication in Dementia Family Caregiving: Using the VNVIS-CG Scale for In-Home Video Observations.

**DOI:** 10.1093/geroni/igab046.1851

**Published:** 2021-12-17

**Authors:** Kristine Williams, Carissa Coleman, Iman Aly, Paige Wilson

**Affiliations:** 1 University of Kansas School of Nursing, Kansas City, Kansas, United States; 2 University of Kansas, Kansas City, Kansas, United States

## Abstract

Communication is fundamental for dementia care and identifying communication behaviors is key to identifying strategies that facilitate or impede communication. To measure caregiver verbal communication, we adapted the Verbal and Nonverbal Interaction Scale for Caregivers (VNVIS-CG) for second-by-second behavioral coding of video observations. The VNVIS-CG was adapted for computer-assisted Noldus Observer coding of video interactions captured at home by family caregivers from the FamTechCare clinical trial. Operational definitions for verbal communication behaviors were developed and inter-rater reliability was excellent (Kappa = .86) using two independent coders. Videos (N=232) were coded featuring 51 dyads; caregivers were primarily female (80%) spouses (69%) of men (55%) diagnosed with moderate to severe dementia (64.7%). Mean caregiver age was 65 years. Silence occurred most frequently (44.9% of the time), followed by caregiver direction or instruction (22.6%), and the person with dementia (PWD) verbalizing (22.8%). Caregiver communication also included asking questions (14.2%), verbalizing understanding (7.9%), repeating information (2.1%), affirmations (1.0%), acknowledging emotions (0.3%), and ignoring (0.0%). Questions most commonly requested clarification, showed interest, or repetitive quizzing; few questions sought to engage PWD input (ex. offers choices, encourages emotional expression, or ask permission). Tone was overwhelmingly neutral rather than humorous, aggressive, or patronizing. The adapted behavioral coding scheme provides a reliable measure that characterizes dementia caregiver verbal communication behaviors for analysis of video observations. Ongoing research will identify strategies that facilitate communication as well as determine how strategies vary by dementia stage, diagnosis, and dyad characteristics.

